# Case report: Clinical and histopathological characteristics of psoriasiform erythema and *de novo* IL-17A cytokines expression on lesioned skin in atopic dermatitis children treated with dupilumab

**DOI:** 10.3389/fmed.2022.932766

**Published:** 2022-07-28

**Authors:** Kamran Ali, Liming Wu, YunMi Qiu, Menghua Li

**Affiliations:** ^1^Department of Dermatology, International Education College of Zhejiang Chinese Medical University, Hangzhou, China; ^2^Department of Dermatology, Affiliated Hangzhou First People's Hospital, Zhejiang University School of Medicine, Hangzhou, China; ^3^Department of Dermatology, Zhejiang Chinese Medical University, Hangzhou, China

**Keywords:** dupilumab, psoriasiform erythema, interlukine-17A, RNA fluorescence *In situ* hybridization, atopic dermatitis (AD)

## Abstract

**Background:**

Atopic dermatitis (AD) is a chronic recurrent inflammatory disease, and dupilumab, a human monoclonal antibody, is the firstly approved biological drug for AD. Psoriasiform erythema (PE) during dupilumab treatment in adults has been reported. This study describes the risk of PE in children after initiation of dupilumab treatment.

**Objectives:**

To evaluate the *de novo* cytokines gene expression in the transition of atopic dermatitis symptoms to psoriasiform erythema during dupilumab treatment in children.

**Methods:**

Two 17-year-old teenage twin patients with AD were included in this study who developed psoriasiform erythema after initiation of dupilumab. The lesional skin biopsy specimens were obtained for the histopathological investigation and RNA Fluorescence *In Situ* Hybridization (RNA-FISH). Dermoscopy, cytometry (cytokine detection in the blood), and blood investigations were completed for the pedigree and the lesioned descriptions.

**Results:**

Two twin patients with AD presented with erythematic scaly plaques on the back, scalp, abdomen, and extensor extremities after 20 weeks of dupilumab treatment. The transitional change of AD to psoriasiform erythema treated with dupilumab was observed. Our subjects' dermoscopy showed pinpoint bleeding and white scales on pink background. Histopathology features showed psoriasiform hyperplasia, epidermal hyperplasia (acanthosis), ectatic capillaries, perivascular lymphocytes infiltration, and parakeratosis, with the absence of the granular cell layer. mRNA (RNA-FISH) cytokines gene expression showed a significantly high concentration of IL-17A. Blood investigation results showed a high concentration of (Immunoglobulin E) IgE and Eosinophils, and cytokines detection in blood showed IL-5,6 and IL-17 in one patient; however, only IL-5 in another patient. The dupilumab was discontinued and initiated with Baricitinib. Baricitinib showed a significant reduction in skin lesions.

**Conclusion:**

Psoriasiform erythema can appear during dupilumab treatment in atopic dermatitis children. Potently, by suppressing skewed Th2 activation in patients with AD, the balance might shift toward Th1/Th17 predominance, and psoriasis develops. Baricitinib is a potential drug for psoriasiform erythema with significant therapeutic effects.

## Introduction

Atopic dermatitis (AD) is a common, chronic type 2 inflammatory skin disease, typically in infancy, increasing the risk of subsequent extracutaneous atopic morbidities (1). Dupilumab is the first Food and Drug Administration-approved human monoclonal antibody blocking interleukin 4 (IL-4) and Interleukin 13 (IL-13) for moderate-to-severe AD. The long-term safety profile of dupilumab is limited due to the biological agents infancy (2, 3). Adverse effects associated with dupilumab have been disclosed in clinical trials, such as conjunctivitis, headache, injection site reaction, exacerbation of atopic dermatitis, and nasopharyngitis (4, 5).

We report on twin patients with AD presenting with erythematic scaly plaques on the back, abdomen, and upper and lower extremities that appeared after 20 weeks of dupilumab treatment. The erythematic plaques were sharply demarcated with silvery scales. Both patients complained of itching and scaling, markedly different from preexisting AD. We highlight the characteristic changes of AD to psoriatic transition.

## Method

This study aimed to describe the development of psoriasiform erythema in children with atopic dermatitis (AD) treated with dupilumab. We conducted the lesion imaging, dermoscopy, completed blood investigations, cytometry (cytokine detection in the blood), histopathology (hematoxylin and eosin), and RNA Fluorescence *In Situ* Hybridization (RNA-FISH) for the pedigree and the lesioned descriptions. Written informed consent was obtained from the guardians/parents.

## Results

**Figure 1:**
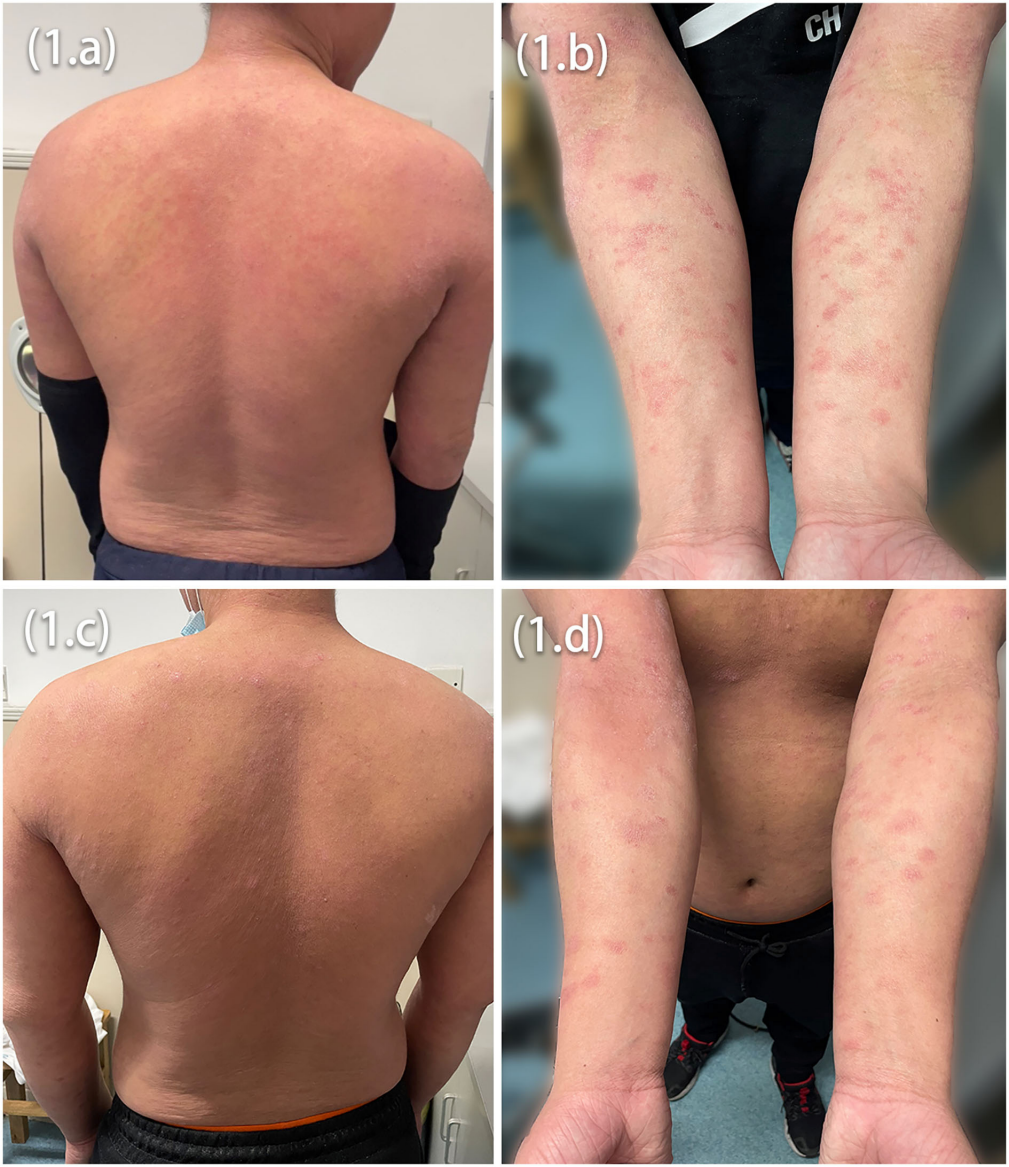
Intensely pruritic, erythematic, lichenified, scaly patches on the trunk **(A,C)** and extensor and flexural surfaces on upper extremities **(B,D)** in both patients before dupilumab treatment initiation.

In our department, more than a hundred (*n* > 100) patients between September 2020 and January 2022 were treated with dupilumab. Two patients developed psoriasiform erythematic lesions. Seventeen-year-old (Asian, Chinese) teenage twin brothers with a history of AD since childhood were diagnosed clinically using Hanifin and Rajka criteria for AD (Figure 1). Patients have intensely pruritic, erythematic, lichenified, scaly patches on the trunk, the head, and the neck, and extensor and flexural surfaces on upper and lower extremities in both patients (Figure 1). They had personal and family history of atopy (allergic rhinitis, asthma). They were refractory to topical corticosteroids, calcineurin inhibitors, phosphodiesterase-4 (PDE4) inhibitors, and systemic therapies (Ebstain, cetirizine).

The patients presented with erythematic scaly plaques on the back, the scalp, the face, the abdomen, and upper and lower extensor extremities after 20 weeks of dupilumab (300 mg, q2w) treatment. The erythematic plaques were sharply demarcated with silvery scales. Both patients complained of itching and scaling, markedly different from preexisting AD.

**Figure 2:**
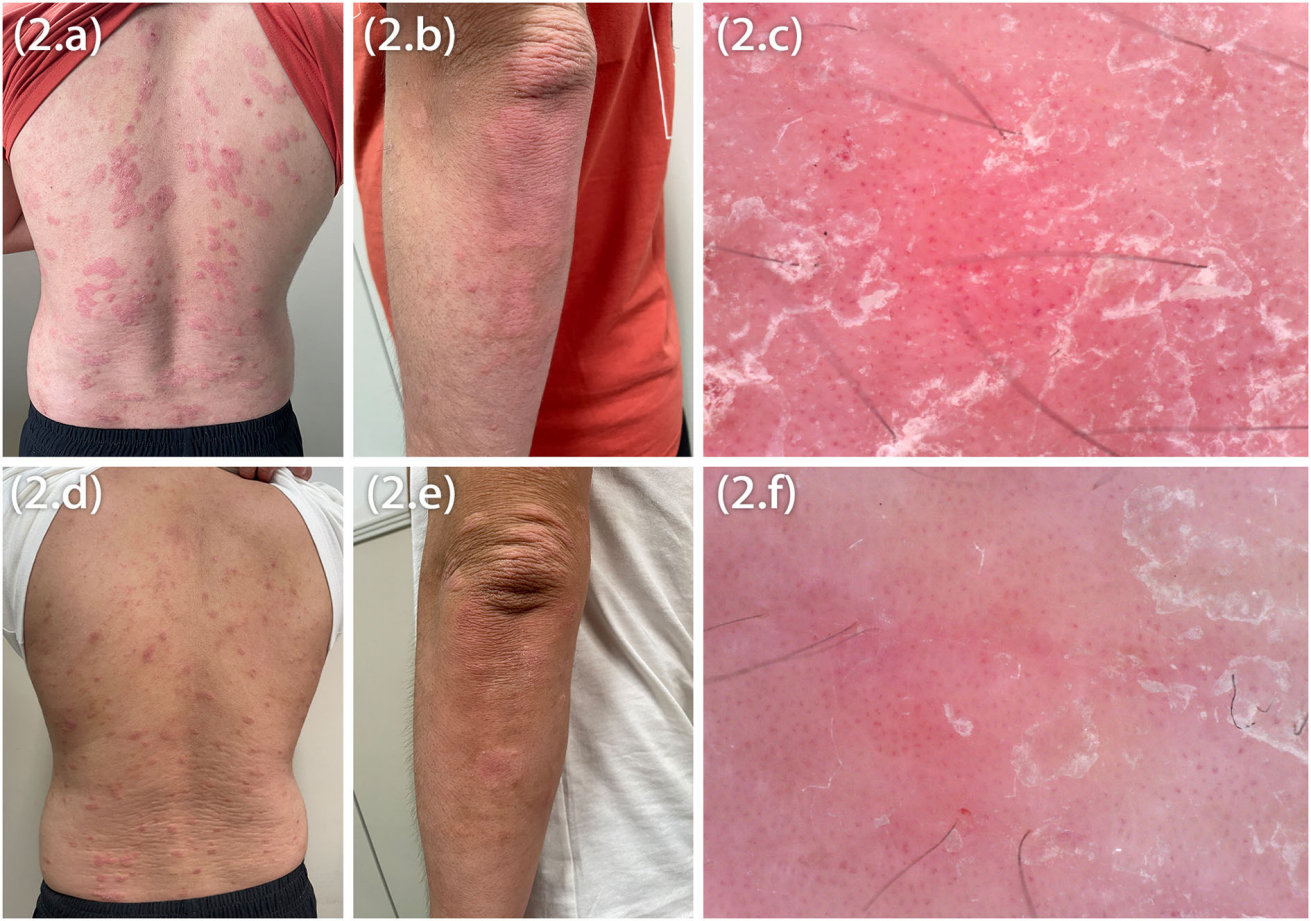
Psoriasiform eruptions on the trunk and the elbow in both patients **(A,B,D,E)**, dermoscopic image showing pinpoint bleeding **(C,F)**.

**Figure 3:**
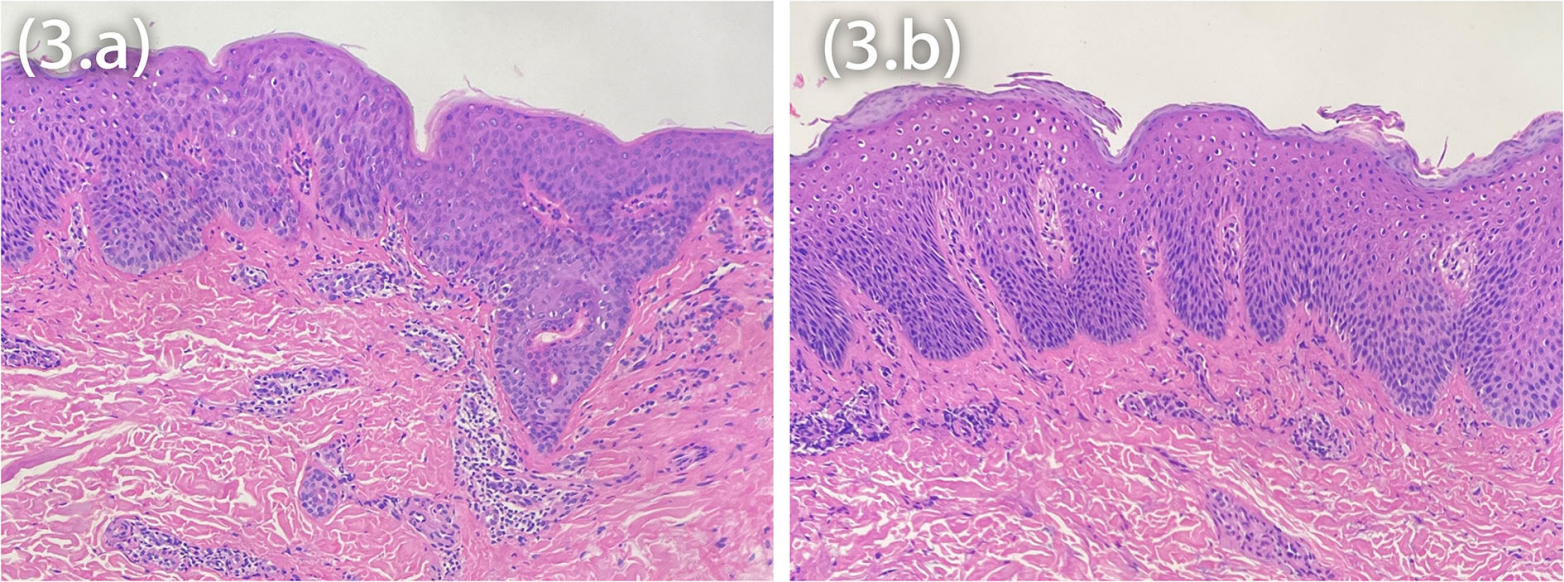
Histopathology showing acanthosis, ectatic capillaries, perivascular lymphocytes infiltration, and parakeratosis with the absence of the granular cell layer **(A,B)**, interestingly the absence of spongiosis.

Marked improvement was observed at Week 12, the average peak pruritus Numerical Rating scale decreased to 1 (NRS = 1), and Eczema Area and Severity Index (EASI 75) was achieved. However, after 20 weeks of dupilumab treatment, the patients observed welldefined erythematic scaly plaques (Figures 2A,B,D,E). The lesions' morphology was highly suggestive of psoriasis. They had no personal or family history of psoriasis but a lifelong history of severe AD that had an inadequate response to topical treatment. There was no history of a recent new infection, sore throat, or fever (white blood cells and neutrophils were in the normal range). Blood investigations showed high IgE and eosinophilia in both patients and elevated IL-6 and IL-17 levels in Patient 1. In our patients, dermoscopy showed pinpoint bleeding and white scales on the pink background (Figures 2C,F). The Auspitz sign was positive for both patients. Paraffin sections were prepared and stained with hematoxylin and eosin. Lesioned histopathological images showed psoriasiform hyperplasia, ectatic capillaries, perivascular lymphocytes infiltration acanthosis with thinning of supra-papillary plates, and confluent parakeratosis with the absence of the granular cell layer consistent with psoriasis. Surprisingly, spongiosis and eosinophils were absent (Figures 3A,B). The dupilumab treatment was discontinued and initiated with baricitinib 2 mg two times daily (bid). The patients showed significant improvement after 16 weeks of treatment. The follow-up was done for 24 weeks by writing this report, and no adverse events were observed. The patients' details are summarized in Table 1.

**Table 1:** Patients and clinical features of atopic dermatitis with psoriasiform eruptions (*n* = 2).

**Patients**	**P1**	**P2**
**Patients characteristics**		
Sex	Male	Male
Age (Years)	17	17
Disease duration	Since childhood	Since childhood
Asthma/Allergic rhinitis/conjunctivitis	Yes family history	Yes family history
Previous treatment	Antihistamine: (Ebstain 10 mg, cetirizine 10 mg), topical calcineurin inhibitors(TCI), topical corticosteroids (TCS), topical phosphodiesterase-4 (PDE4) inhibitors	Antihistamine: (Ebstain 10 mg, cetirizine 10 mg), topical calcineurin inhibitors(TCI), topical corticosteroids (TCS), topical phosphodiesterase-4 (PDE4) inhibitors
Onset of Psoriasiform eruptions (weeks of dupilumab treatment)	20 weeks	20 weeks
Treatment duration at biopsy	24 weeks	24 weeks
Blood investigations	IL-17: ↑32.6 pg/ml, IL-5: ↑57.2 pg/ml, IL-6: ↑10.8 pg/ml, IL-4: 2.3 pg/ml, TNF-a: <0.8 pg/ml, Eosinophil: 0.43 * 103 /uLIgE: 9572.4 IUmL WBC: 8.53 * 10^3^/uL Neutrophil: 1.19 * 10^3^/uL	IL-17: 3.2 pg/ml, IL-5: ↑29.7 pg/ml, IL-6: 2.2 pg/ml, TNF-a: <0.7 pg/ml, IL-4: 2.8 pg/ml, Eosinophil:0.63 * 10^3^ /uL, IgE: 4890.6 IUmL WBC: 8.36 * 10^3^/uL Neutrophil: 5.22 * 10^3^/uL
Symptoms of Psoriasiform eruptions	Hyperkeratotic well-defined erythematic skin lesions appeared on the upper and lower extremities, scalp, trunk, back, and abdomen. Auspitz sign: + Itching: +	Erythematous and scaly plaques on trunk, back, scalp, abdomen, bilateral upper and lower limbs. Auspitz sign: + Itching: +
Topical treatment	Tacrolimus 0.1% and cetaphil moisturizing lotion	Tacrolimus 0.1% and cetaphil moisturizing lotion
Systemic prescriptions	Baricitinib (2 mg twice a day)	Baricitinib (2 mg twice a day)

*P1, patient 1; P2, patient 2; TCI, topical calcineurin inhibitors; TCS, topical corticosteroids; IL, interleukin; TNF-a, Tumor necrosis factor; IgE, Immunoglobulin E; +, positive; WBC, white blood cells*.

## Discussion

Since childhood, both patients have had AD and were treated with dupilumab for 6 months—the patients developed psoriasiform erythema, distributing the scalp, the face, the abdomen, extensor upper and lower extremities, and the back. The histopathological features include parakeratosis without the granular cell layer, acanthosis, dilated capillaries, psoriasiform hyperplasia, ectatic capillaries, and lymphocytic infiltration, which were observed in the upper dermis of the biopsy taken from the patients' back and abdomen, respectively. Interestingly, the histopathology findings showed the absence of eosinophils and spongiosis in both patients. Clinical manifestations and histopathological findings were not characteristics of AD. The histopathological features of acute AD are spongiosis, perivascular lymphocytic, and eosinophilic infiltration. Acanthosis can be seen in subacute and chronic AD lesions, occasionally in a psoriasiform pattern; however, hyperkeratosis, fibrosis, spongiosis, thick infiltrates of mononuclear cells, eosinophils, and increased mast cells are characteristics of subacute and chronic AD. Surprisingly, we discovered that the histological features of atopic dermatitis were missing. Spongiosis was also absent, as were mononuclear, mast cell and eosinophilic infiltrates, confirming our clinical assessment that this was not typical atopic dermatitis (6). Psoriasiform lesions are not common among children or adults who take dupilumab. However, due to the clinical appearance of lesions and the temporal nature of their occurrence, the possibility exists that they are related to dupilumab therapy.

Recently, some adult case reports have been reported; Tracey et al. presented a case of erythrodermic psoriasis in a woman in her 50 s (7), Gori et al. reported a case of guttate psoriasis (8), Stout et al. presented a case of psoriatic-like lesions during dupilumab treatment (9). Fowler et al. discussed a case series of psoriasiform dermatitis in two patients after dupilumab initiation and confirmed by the skin biopsies (10). Jennifer et al. reported a case series of six children with moderate-to-severe atopic dermatitis who developed a new onset of psoriasiform dermatitis. However, the histopathological investigations were not performed on any of the patients (11).

Psoriasis and atopic dermatitis (AD) are systemic T-helper (Th) cellular-driven inflammatory skin disorders characterized by different cytokine pathways. Psoriasis is mainly driven by T-helper 17 (T-cells) and concomitant interleukine-17 (IL-17) activation. In contrast, AD has a robust Th2 component associated with interleukine-4 (IL-4) and interleukine-13 (IL-13) overproduction and activation of T-helper 22 (Th22) T-cells, T-helper 1 (Th1) pathways with increased IL-22, and Tumor necrosis factor-alpha (TNF-α) production in both disorders, respectively. Atopic dermatitis is usually accompanied by increased IgE production and overt allergies or asthma, most likely due to enhanced Th2 activation, which is absent in psoriasis (12). It has been demonstrated that lymphocyte subset balance is essential in inflammatory skin disorders. Inhibiting subsets of cytokines to treat such disorders can create an imbalance that favors other subsets (12). As dupilumab is a monoclonal antibody inhibiting IL-4/IL13 signaling and downregulating the Th2 inflammatory response, Dupilumab's inhibition of the Th2 pathway hypothetically results in activating the Th1 pathway. Conversely, patients with psoriasis or other Th1-mediated diseases may develop Th2-mediated disease after being treated with Th1-inhibiting agents. Furthermore, treating the psoriatic patient with an IL-17 inhibitor can induce eczematous reactions (13).

**Figure 4:**
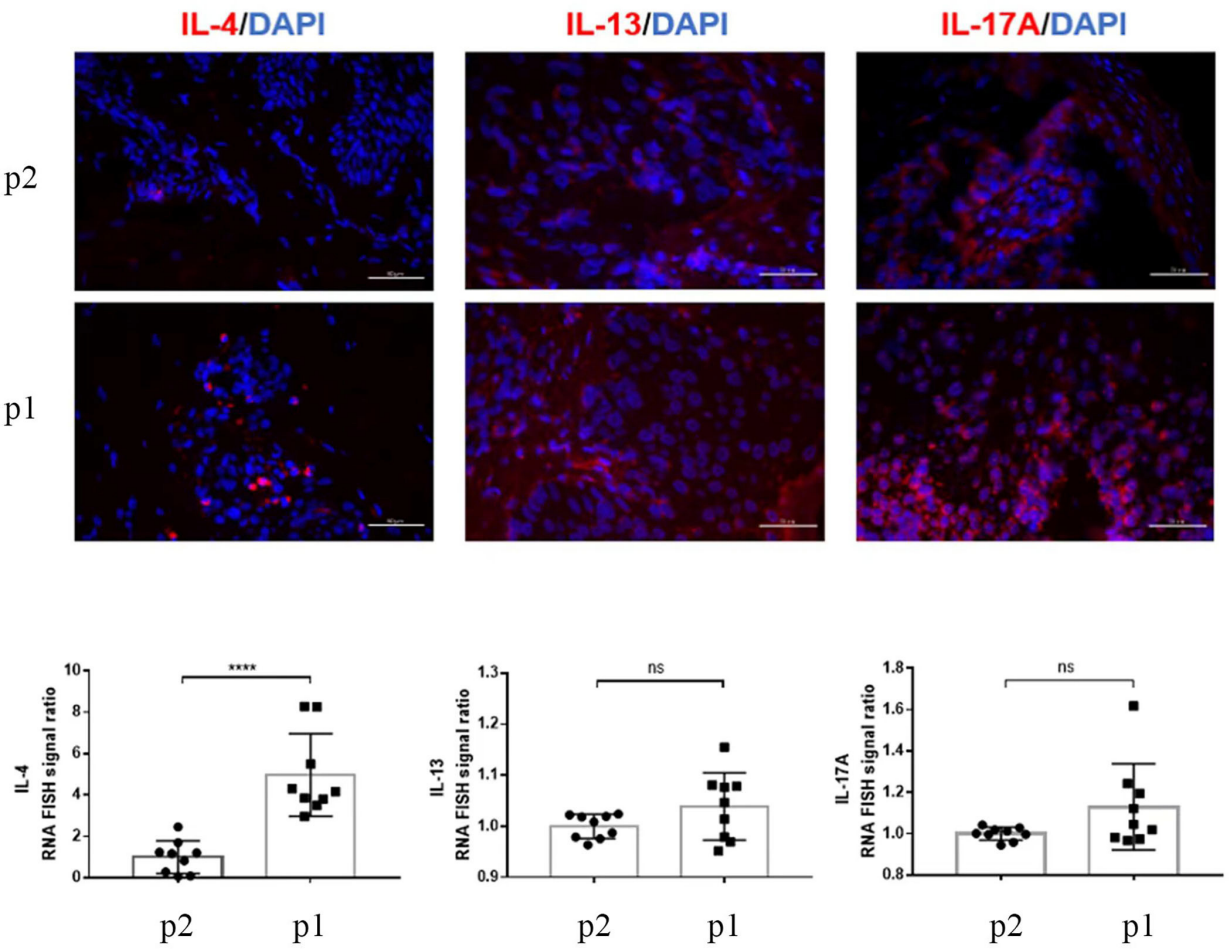
RNA-FISH images show the expression of IL-4, IL-13, and IL-17A (red) in skin tissue sections P2 (Patient 2) and P1 (Patient 1). More than nine random areas on each slide were examined, and representative images were shown. Scale bars are 50 μm. The quantification of nine images is shown, and data are presented as mean values ± SD. The positive RNA-FISH signals were analyzed using the ImageJ software. Statistical analyses were conducted using GraphPad Prism 7. * * * * *p* < 0.0001, ns: *p* > 0.05.

The high levels of TNF-α, IL-6, and IL-17 cytokines are involved in psoriasis's pathogenesis. Interestingly, one of our patients had elevated IL-6 and IL-17 levels, resulting in the psoriasiform reaction pattern, but the TNF-α was in the normal range. Furthermore, our patients also had high IgE and eosinophilia, primarily high concentrations in AD, as we found the heterogeneity in the blood investigation and clinical lesions. mRNA-FISH for IL-4, IL-13, and IL-17A gene expression was compared in twin brothers. Moreover, a large amount of the IL-17A and IL-13 mRNA-FISH signal ratio was detected (^*^^*^^*^^*^ : *p* < 0.0001, ns: *p* > 0.05) (Figure 4). A case study reported that a patient with AD who received phototherapy and topical corticosteroid therapy failed and switched to dupilumab to continue the treatment. Psoriasis-like plaques appeared on the extremities 8 months later. Through immunohistochemical experiments on skin biopsy plaques, the expression levels of IL-4, IL-13A, IL-12B, IL-17A, and IL-23A in psoriasis-like plaques of patients with AD before and after dupilumab treatment were compared. The results showed that patients with AD treated with dupilumab had new psoriasis skin lesions, with a large amount of new IL-17A production and a small amount of IL-23A upregulation (14). Interestingly, a high level of IL-17A gene expression in our patients was also observed in mRNA-FISH testing and was supporting our diagnosis. We mainly focus on analyzing the changes in expression of IL-4, IL-13, and IL-17A after treatment, therefore calculating the fold relationship of corresponding fluorescence intensity between Patient 1 and Patient 2. The results were compared to the clinical presentation. It is known that AD may present Th17-related cytokines, especially in pediatric and Asian patients (15). The results of our study are more descriptive than comparison. However, our patients were teenagers diagnosed using Hanifin and Rajka criteria for AD; histopathological samples were not taken before treatment. So, the lesional cytokine expression was not performed before dupilumab treatment, which is the limitation of this study. Nevertheless, they had no personal or family history of psoriasis, but they had a lifelong history of severe AD and personal and family history of comorbidities (allergic rhinitis and asthma).

After discussion with guardians, our patients were treated off-label with a baricitinib 2 mg bid and showed a significant decrease in the disease severity. Baricitinib is an oral selective small-molecule Janus Kinase (JAK) 1 and 2 inhibitors (16). In 2018, baricitinib was approved to treat rheumatoid arthritis. Current phase III trials are being conducted for treating atopic dermatitis as well as phase IIb trials for the treatment of moderate-to-severe psoriasis (NCT01490632) (17–20).

Further studies are required to identify the subset of patients predisposed to this *de novo* cytokine gene expression to confirm the shift from Th2 to Th1/Th17 and to understand the pathogenesis and the safety and effectiveness of JAK inhibitor therapies for psoriasiform erythema in teenagers.

## Conclusion

This study presents the onset of psoriasis in Chinese teenage patients with AD treated with dupilumab with detailed clinical and histopathological characteristics and mRNA-FISH cytokines expression on skin lesions. This suggests that these erythematic scaly plaques were a dupilumab-induced reaction and highlights the importance of practitioners learning about the incidence of this condition. Incorporating mRNA-FISH in patients with AD may help further understand the heterogeneity and pathogenesis of these psoriatic lesions caused by dupilumab. Baricitinib showed significant therapeutic effects on these patients. However, more studies are needed to understand the underlying mechanism of this phenomenon.

## Data availability statement

The original contributions presented in the study are included in the article/supplementary material, further inquiries can be directed to the corresponding author/s.

## Ethics statement

Written informed consent was obtained from the participants/guardians for the publication of any potentially identifiable data or images presented in the manuscript.

## Author contributions

KA: conception, design of the study, and writing the original manuscript. LW: provided article ideas and final approval of the version to be submitted. ML: analysis and interpretation of data. YQ: drafting the article or revising it critically for important intellectual content.

## Conflict of interest

The authors declare that the research was conducted in the absence of any commercial or financial relationships that could be construed as a potential conflict of interest.

## Publisher's note

All claims expressed in this article are solely those of the authors and do not necessarily represent those of their affiliated organizations, or those of the publisher, the editors and the reviewers. Any product that may be evaluated in this article, or claim that may be made by its manufacturer, is not guaranteed or endorsed by the publisher.
